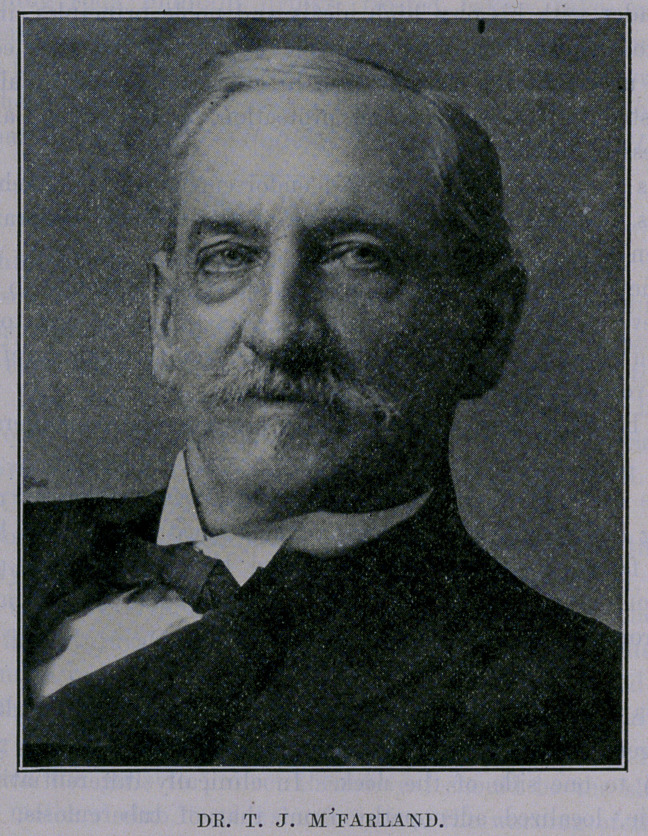# Death of Dr. McFarland

**Published:** 1914-04

**Authors:** 


### EDITORIAL DEPARTMENT.

**DR. F. E. DANIEL, Editor** **DR. R. H. L. BIBB, Associate Editor**

DEPARTMENTS { EUGENICS: Drs. M. Duggan and T. Y. Hull.

WOMAN'S DEPARTMENT: Mrs. F. E. Daniel.

### Death of Dr. McFarland.

One by one the typical physicians of the Old South are passing away. Their ranks are rapidly thinning, and in a few years the type will have become extinct; the aristocratic physician of long ago will be but a memory, and mark but a phase in a passing

DR. T. J. M'FARLAND.

Died, at his home in Lavaca, Texas, February 24, 1914, Dr. Thomas J. McFarland, in the seventy-eighth year of his age.

civilization. It is a distinct type, characterized by dignity, learning, culture, innate courtesy, chivalry, and devotion to the profession. Dr. McFarland was a splendid specimen of his class, physically as well, and in every relation of life was an exceptional man. He is numbered in my annals with the beloved Swearingen, Paine, T. C. Osborn, Cupples, McLaughlin, the great S. H. Stout, the Confederate hospital director, and D. R. Wallace. I knew him well,—graduated in the same class with him fifty odd years ago, and with him entered the medical service of the Confederate army. That I should love him is but natural. No one could help loving him who came within the charm of his presence, his soft voice, gentle eyes and genial smile. I do not regret him. He has gone to his reward for a life well spent in the cause of humanity, and he has left a family of splendid children, fine strong men and gracious, charming women, wives and mothers.

"Green grow the turf above thee,
Friend of my better days.
None knew thee but to love thee,
None named thee but to praise."

BIOGRAPHICAL.

Dr. McFarland was born in Alabama, July 1, 1836. He graduated with high honors from Jonesboro, Alabama, College and removed to Mississippi in his youth, settling in Rankin county. There he took up the study of medicine and attending lectures at the old University of Louisiana, now Tulane, and also the New Orleans School of Medicine. He graduated from the latter in February, 1862. Immediately he went before the army board of medical examiners of the Army of Tennessee—Yandell, Pim and Heustis—and was commissioned by the Secretary of War, surgeon (major). He was assigned to a regiment in Bragg's army, but rose to brigade surgeon. He served all through the war, the latter part of his service being in the hospitals under the great S. H. Stout, the only medical director of hospitals we ever had.

On August 3, 1864, while still on duty in the army, Dr. McFarland was married at Brandon, Miss., to Carrie P. Jayne, of the very distinguished Jayne family of Mississippi, and took his bride to the city of Columbus, Georgia, where he had charge of the army post hospital. Coming to Texas he located at old Indianola in the service of the United States marine service as government quarantine officer. Here he had a most harrowing experience when Indianola was destroyed by storm in 1873. In the night and darkness he found his house afloat and the waters up to the second story. He fled with wife and four small children to the highest point, and when a wave would deluge them he and his wife would have to hold the little fellows up. In the morning when the storm had subsided it was found that his house was stranded on a sand bar a mile out. That was the last of Indianola—all washed away. Dr. McFarland then settled at Port Lavaca, where he resided and engaged in the active practice of medicine up to the day of his death. He leaves besides his widow, six grown children: Dr. Van E. McFarland, El Paso, Texas; Minter McFarland, Alpine, Texas; Carl McFarland, Fort Worth, Texas; Mrs. Frank Penfield, El Campo, Texas; Mrs. Dr. Thompson Grace, Mrs. F. M. Dudgeon, Miss Belle McFarland, Port Lavaca, Texas.

## Figures and Tables

**Figure f1:**